# Long-Term Outcome of Rectal Cancer With Clinically (EUS/MRI) Metastatic Mesorectal Lymph Nodes Treated by Neoadjuvant Chemoradiation: Role of Organ Preservation Strategies in Relation to Pathologic Response

**DOI:** 10.1245/s10434-016-5451-5

**Published:** 2016-08-03

**Authors:** Claudio Belluco, Marco Forlin, Matteo Olivieri, Renato Cannizzaro, Vincenzo Canzonieri, Angela Buonadonna, Ettore Bidoli, Fabio Matrone, Giulio Bertola, Antonino De Paoli

**Affiliations:** 1 Department of Surgical Oncology, CRO–IRCCS, National Cancer Institute, Aviano, Italy; 2 Department of Gastroenterology, CRO–IRCCS, National Cancer Institute, Aviano, Italy; 3 Department of Pathology, CRO–IRCCS, National Cancer Institute, Aviano, Italy; 4 Department of Medical Oncology, CRO–IRCCS, National Cancer Institute, Aviano, Italy; 5 Department of Epidemiology, CRO–IRCCS, National Cancer Institute, Aviano, Italy; 6 Department of Radiation Oncology, CRO–IRCCS, National Cancer Institute, Aviano, Italy

## Abstract

**Background:**

Organ preservation strategies are under investigation for patients with locally advanced rectal cancer (LARC) who achieve a complete pathologic response in the primary tumor (ypT0) after neoadjuvant chemoradiation therapy (CRT). This study explored the value of this approach for cN+ patients.

**Methods:**

Data were retrieved from our institutional prospective rectal cancer database. Tumors with mesorectal lymph nodes larger than 5 mm shown on endorectal ultrasonography, pelvic magnetic resonance imaging, or both were staged as cN+.

**Results:**

The study population comprised 226 patients (142 men and 84 women; median age, 64 years) with LARC who underwent CRT followed by surgery including total mesorectal excision (TME) (*n* = 179) and full-thickness local excision (LE) (*n* = 47) between 1996 and 2013. At staging, 123 patients (54.4 %) were cN+. In 65 cases (28.7 %), ypCR was observed. Metastatic mesorectal lymph nodes (ypN+) were detected in 41.6 % of the cN+ patients and in 2.8 % of the cN0 patients (*P* < 0.01). Among the cN+ patients, 16 % of the ypT0 cases were ypN+ compared with 51.8 % of the no-ypT0 cases (*P* < 0.01). Among the cN+ patients who underwent TME, the 5-year disease-specific survival (DSS) and disease-free survival (DFS) rates were respectively 100 and 91.6 % for the ypT0 patients compared with 71.2 and 58.0 % for the no-ypT0 patients (*P* = 0.01). Among the ypN+ patients, the 5-year DSS and DFS rates were both 100 % for the ypT0 cases compared with 59.1 and 43.3 % for the no-ypT0 patients. Among the cN+ and ypT0 patients, the 5-year DSS and DFS were respectively 100 and 85.7 % for the TME patients compared with 100 and 91.6 % for the LE patients. In the multivariate analysis, ypT0 was the only independent prognostic factor.

**Conclusions:**

Protocols aimed at organ preservation in LARC that achieve ypT0 after CRT can be offered also to cN+ patients.

Neoadjuvant chemoradiation therapy (CRT) and radical surgery including total mesorectal excision (TME) reduces the risk of local recurrence and is considered the standard of care for patients with locally advanced (T3–4 or any N1–2) mid-distal rectal cancer (LARC).^1^^–^ 4 A pathologic complete response (ypCR) shown in the surgical specimen of LARC patients treated by CRT is observed in up to one-third of the cases.^5^

In ypCR cases, a favorable long-term oncologic outcome has been observed,^6^^–^ 9 and organ preservation strategies including transanal full-thickness local excision and/or close observation are being explored in patients displaying clinical or pathologic complete response to CRT. This would lead to a reduction in surgery-related morbidity and mortality and to quality-of-life improvement.^10^^–^ 23

The potential presence of metastatic mesorectal lymph nodes, with the related risk of local and distant recurrences, represents a key limiting factor for the application of organ preservation strategies. The reported rate of metastatic mesorectal lymph nodes in the surgical specimen of LARC patients achieving a complete pathologic response (ypT0) in the primary tumor is variable,^24^^–^ 33 and the accuracy of lymph node status restaging after CRT is low.^34^^–^ 36

Because a priori knowledge of pathologic and oncologic outcome risks is an important issue for protocol design and for clinician–patient communication at clinical study enrollment, we specifically focused this study on patients with rectal cancer staged by endorectal ultrasonography (EUS), pelvic magnetic resonance imaging (MRI), or both as having metastatic mesorectal lymph nodes at their initial diagnosis (cN+).

To evaluate whether cN+ patients could be reasonably eligible for treatment strategies aimed at organ preservation, we analyzed the pathologic and long-term oncologic outcomes for LARC patients treated by neoadjuvant CRT at our institution during a 17-year period.

## Methods

All consecutive informed-consent patients treated by neoadjuvant CRT and surgery for LARC between January 1996 and October 2013 were identified from our institutional, prospectively maintained, rectal cancer database. Patients with synchronous distant metastasis were excluded from the study.

All the patients had biopsy-proven adenocarcinoma of the rectum. The distance of the tumor from the anal verge was measured by rigid rectoscopy. Pre- and post-CRT primary tumor and nodal stagings were evaluated by EUS, pelvic MRI, or both. Lymph nodes 5 mm or larger were considered positive. In cases with discrepancy between the two imaging techniques, the higher stage was considered. Distant metastases were ruled out by thoracoabdominal and pelvic CT scan.

### Treatment

#### Preoperative CRT

Preoperative CRT was administered according to several preoperative sequential treatment protocols developed at our institution, including a 5-fluorouracil (5-FU) bolus + leucovorin (LV) and 45 Gy with or withut adjuvant 5-FU/LV, raltitrexed and 50.4 + 10 Gy of intraoperative radiation therapy (IORT), capecitabine and 50.4 Gy, continuous infusion 5-FU + gefitinib and 50.4 + 10 Gy IORT, and capecitabine ± oxaliplatin and 50.4 Gy. The radiotherapy (RT) clinical target volume (CTV2) included the primary tumor, the mesorectum, and internal iliac lymph nodes. A second clinical target volume (CTV1) included the mesorectum corresponding to the primary tumor with a 2-cm radial margin. The RT fractionation was 180 cGy/day, 5 fractions per week. More details on RT technique and dose prescription have been reported previously.^8^

#### Surgery

The patients underwent surgery 6–8 weeks after completion of neoadjuvant CRT. The surgical procedures included abdominoperineal resection (APR), low anterior resection (LAR), and full-thickness transanal local excision (LE). Radical resection was performed according to TME principles. Reasons for the use of LE included medical comorbidity and patient refusal of APR for low-lying tumors not eligible for coloanal reconstruction due to anticipation of poor sphincter function.

In more recent years, patients with a major clinical response to CRT were offered the option of LE in a prospective clinical study investigating the outcome of LE after a complete clinical and pathologic response. In these cases, LE was used to assess the pathologic response in the primary tumor. Medically fit patients showing no complete or almost complete pathologic response in the primary tumor (TRG1 and TRG2 according to Mandard tumor response grading)^37^ underwent subsequent TME surgery. After surgical resection, IORT to a high risk area (presacral region) was administered according to study protocols, as mentioned earlier.

#### Postoperative Chemotherapy

Adjuvant 5-FU-based chemotherapy was administered according to the study protocol, or in selected cases included patients with metastatic lymph nodes.

### Pathology

Pathologic tumor staging was performed according to the guidelines of the American Joint Committee on Cancer and the College of American Pathologists.^38^ Patients with no residual cancer cells in the surgical specimen were considered pathologic complete responders (ypCR).

### Follow-up Evaluation

Postoperatively, the patients were examined at follow-up visits every 3 months for the first 2 years and half-yearly thereafter. At each follow-up control visit, the CEA level was determined. Abdominal and pelvic computed tomography (CT) scan or liver ultrasound and chest x-ray were performed alternatively every 3–6 months. Colonoscopy was performed yearly.

### Statistical Analysis

The Chi square test or Fisher’s exact test was used to compare percentages between complete responders and non-complete responders, and the Wilcoxon rank test was used for median age comparison. Cumulative probabilities of overall survival (OS), disease-specific survival (DSS), disease-free survival (DFS), distant metastasis-free survival (DMFS), and local recurrence-free survival (LRFS) were estimated by Kaplan–Meier survival methods,^39^ and differences between subgroups were assessed using the log-rank test. The duration of follow-up evaluation was calculated as the time from surgery to the event of interest. Patients without event were censored at the date of the last follow-up visit. In cases with local and distant metastasis, both events were recorded and computed at any time of occurrence. For better assessment of the oncologic implications of ypCR, the Cox proportional hazards model was used to adjust the hazard ratios (HRs) and corresponding 95 % confidence intervals (CIs).^40^ Due to the limitation of sample size and number of events, only three variables were entered into the multivariate model: cNstage (cN0 vs cN1), type of surgery (TME vs LE), and ypT0 (yes vs no). Collinearity between variables was excluded by means of the Chi square test. A *P* value of 0.05 or lower was considered statistically significant (two-tailed). The SAS System 9.2 (SAS, Cary, NC, USA) was used as the statistical software for data analysis.

## Results

### Patients and Treatment Characteristics

The study population comprised 226 consecutive patients (142 men and 84 women; median age, 64 years; range, 25–87 years) with mid-distal LARC and no distant metastasis treated by neoadjuvant CRT followed by surgery at our institution between January 1996 and October 2013.

At the initial evaluation, 226 patients were staged as follows: 5 cT2N1 (2.2 %), 79 cT3N0 (34.9 %), 104 cT3N1 (46 %), 12 cT4N0 (5.3 %), 13 cT4N1 (5.7 %), 2 cTxN0 (0.8 %), and 1 cTxN1 (0.4 %). In addition, 10 very low-lying cT2N0 tumors (4.4 %) were considered at high risk for recurrence, treated by neoadjuvant CRT, and included in this study. The median distance of the tumor from the anal verge was 5 cm (range, 1–12 cm). The total RT dose was 45 Gy for 42 patients (18.5 %), 50.4 Gy for 180 patients (79.6 %), and 25 Gy for 4 patients (1.7 %). Total mesorectal excision was performed for 179 patients (79 %) (142 LAR and 37 APR), whereas LE was performed for 47 patients (21 %). The documented reasons for the use of LE were preference after a major clinical response in 22 cases, patient absolute refusal of APR in 4 cases, and medical comorbidity in 3 cases. The remaining 18 patients were enrolled in the prospective clinical study investigating the outcome for LE after complete clinical and pathologic response. All patients restaged as ycN+ (*n* = 24) underwent TME surgery. Intraoperative radiation therapy was applied in the context of clinical studies. Postoperative chemotherapy was administered to all 33 ypN+ patients (14 %).

### Clinical and Pathologic Response

In the entire patient population, a complete pathologic response in the primary tumor (ypT0) was observed in 65 cases (28.7 %). For the 179 patients who underwent TME, the ypCR rate (ypT0N0) was 20.1 % (*n* = 36). The median number of examined lymph nodes was 13 (range, 2–37). Metastatic lymph nodes (ypN+) were found in 47 (26.2 %) of the surgical specimens: in 4 (10 %) of 40 ypT0 cases, in 1 (9 %) of 11 ypT1 cases, in 12 (21 %) of 57 ypT2 cases, in 28 (43 %) of 65 ypT3 cases, and in 2 (33.3 %) of 6 ypT4 cases.

Among the patients who underwent TME surgery, metastatic lymph nodes (ypN+) were detected in 45 (41.6 %) of 108 cN+ patients compared with 2 (2.8 %) of 71 cN0 patients (*P* < 0.01).

In the subgroup of cN+ tumors with ypT0 treated by TME surgery, 4 (16 %) of 25 (all restaged as ycN0) were ypN+ compared with 43 (51.8 %) of 83 cases that had no ypT0 (*P* < 0.01).

In the subgroup of cN0 tumors with ypT0 treated by TME surgery, 0 of 15 were ypN+ compared with 2 (3.5 %) of 56 no-ypT0 cases.

At restaging after CRT, comparing ycN status with ypN status, metastatic lymph nodes at pathology were detected in 9 of 23 ycN+ cases and in 10 of 25 ycN0 cases (sensitivity, 0.47; specificity, 0.51).

### Recurrence and Survival

No postoperative mortality occurred. During a median follow-up period of 48 months, 20 patients (8.84 %) experienced local recurrence only, 14 (6.19 %) experienced local recurrence and distant metastasis (9 liver, 4 lung and 1 other site cases), and 34 (15.04 %) experienced distant metastasis only (15 liver, 9 lung, 7 liver and lung, and 3 multiple-site cases).

In the comparison of ypCR-patients (ypT0N0) and no-ypCR patients who underwent TME surgery, the 5-year DSS was respectively 91.0 and 79.4 % (*P* = 0.029), and the 5-year DFS was 84.9 and 61.7 % (*P* = 0.011).

In the entire patient population, the 5-year survival rates were 79.2 % for OS, 83.0 % for DSS, 66.9 % for DFS, 77.1 % for DMFS, and 82.6 % for LRFS. In the subset of 65 ypT0 patients, 2 (3.1 %) experienced local recurrence only, 3 (4.61 %) experienced local recurrence and distant metastasis, and 3 (4.61 %) experienced distant metastasis only (1 liver, and 2 liver and lung cases).

In the comparison of ypT0 patients (*n* = 65) with no-ypT0 patients (*n* = 161), the 5-year OS was respectively 89.4 versus 75.3 % (*P* = 0.005), the 5-year DSS was 94.5 versus 78.4 % (*P* = 0.005), the 5-year DFS was 87.3 versus 58.8 % (*P* < 0.001), the 5-year DMFS was 93.0 versus 70.6 % (*P* = 0.002), and the 5-year LRFS was 90.5 versus 79.3 % (*P* = 0.034). According to the clinical lymph node status at initial diagnosis, the 5-year DSS and DFS were respectively 80.5 and 64.2 % in cN+ cases compared with 86.3 and 69.7 % in cN0 cases (nonsignificant difference) (Table 1).

**Table 1:** Long-term oncologic outcome according to clinocopathologic characteristics in locally advanced rectal cancer patients treated by neoadjuvant chemoradiation

Variable	Total	5-yrs DSS		5-yrs DFS		5-yrs LRFS	
	*n* (%)	%	*P* Value	%	*P* Value	%	*P* Value
Sex							
Female	84 (37.2)	80.2	0.638	69.9	0.461	89.7	0.042
Male	142 (62.8)	84.4		65.1		78.0	
Age (years)							
≤65	128 (56.6)	82.2	0.863	67.5	0.896	82.8	0.874
>65	98 (43.4)	84.0		65.9		82.1	
cN status							
cN0	103 (45.6)	86.3	0.537	69.7	0.537	83.0	0.872
cN+	123 (54.4)	80.5		64.2		82.2	
Type of surgery							
TME	179 (79.2)	81.8	0.201	66.4	0.700	84.0	0.312
LE	47 (20.8)	87.2		68.2		76.8	
ypT status							
ypT0	65 (28.7)	94.5	0.005	87.3	<0.001	90.5	0.034
No ypT0	161 (71.3)	78.4		58.8		79.3	
ypN status				66.2		80.9	
ypN0	132 (73.8)	88.0	0.002	72.2	0.008	85.4	0.309
ypN+	47 (26.2)	71.7		55.7		85.7	

*DSS* disease-specific survival, *DFS* disease-free survival, *LRFS* local-recurrence-free survival, *cN* clinical lymph node, *TME* total mesorectal excision, *LE* local excision, *ypT0* complete pathologic response in the primary tumor, *ypN* pathologic lymph node status (only TME patients)

**Fig. 1:**
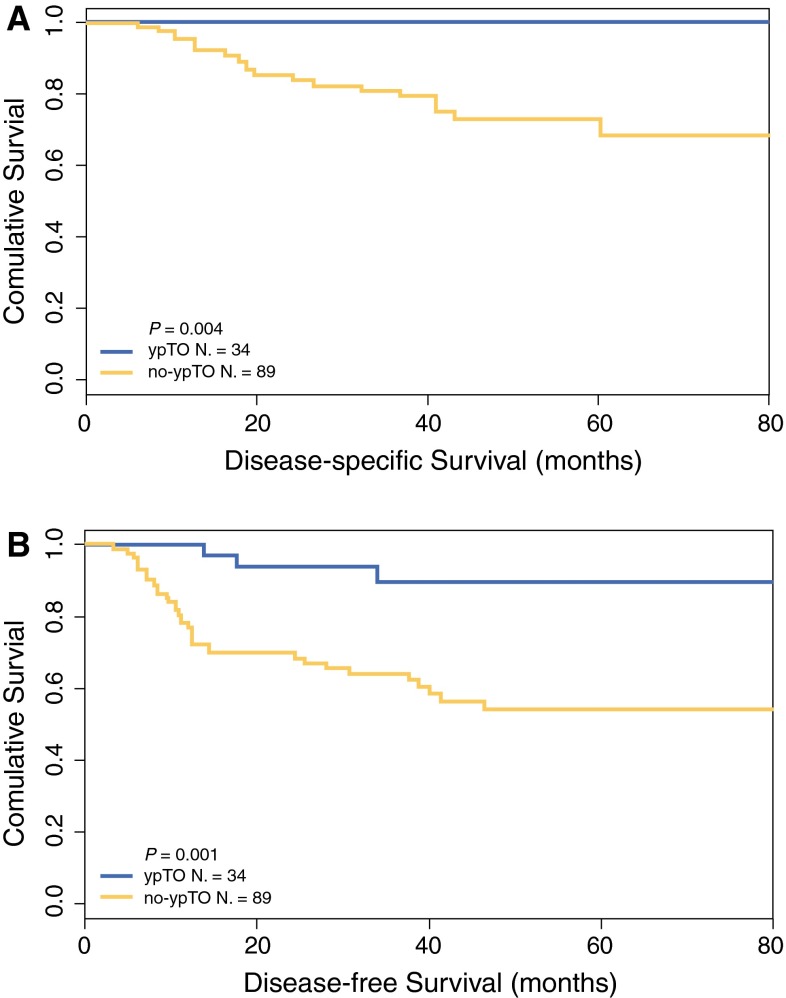
Kaplan-Meier estimates for disease-specific survival (**a**) and disease-free survival (**b**) according to a complete pathologic response of the primary tumor (ypT0) in 123 cN+ rectal cancer patients treated by neoadjuvant chemoradiation followed by total mesorectal excision (TME) or full-thickness local excision (LE) surgery

**Fig. 2:**
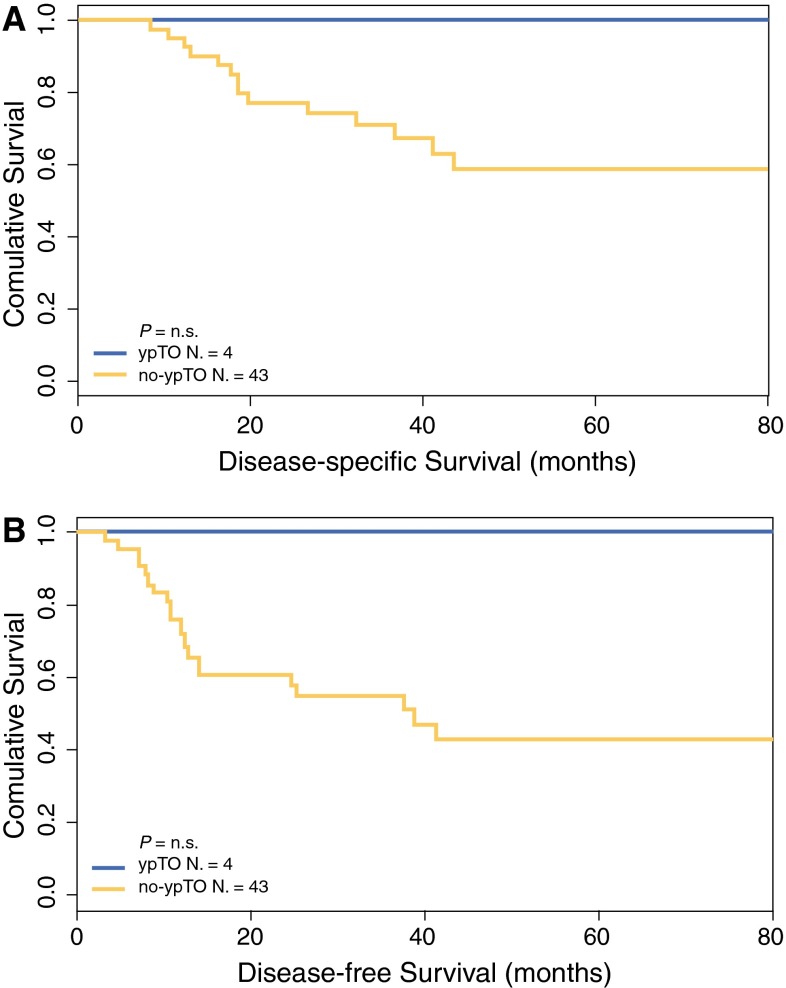
Kaplan-Meier estimates for disease-specific survival (**a**) and disease-free survival (**b**) according to a complete pathologic response of the primary tumor (ypT0) in 47 ypN+ rectal cancer patients treated by neoadjuvant chemoradiation followed by total mesorectal excision (TME) surgery

Among the cN+ patients (*n* = 123) the 5-year DSS and DFS were respectively 100 and 91.6 % for the ypT0 patients compared with 71.2 and 58.0 % for the no-ypT0 patients (*P* < 0.01; Fig. 1). The 5-year DSS and DFS were both 100 % for the 4 ypT0N+ patients compared with 59.1 and 43.3 % respectively for the 43 no-ypTN+ patients (nonsignificant difference; Fig. 2).

Among the cN+ patients who achieved ypT0, the 5-year DSS and DFS were respectively 100 and 85.7 % for the TME patients (*n* = 108) compared with 100 and 91.6 % for the LE patients (*n* = 15) (nonsignificant difference). In the multivariate analysis, ypT0 was the only independent prognostic factor for DSS (HR, 0.13; 95 % CI, 0.03–0.58; *P* = 0.007) and for DFS (HR, 0.25; 95 % CI, 0.12–0.54; *P* < 0.001).

## Discussion

The current study investigated whether LARC patients initially staged as cN+ and achieving ypT0 after neoadjuvant CRT are potential candidates for organ-preserving surgical strategies. To this end, the rate of ypT0, the incidence of metastatic lymph nodes, and the long-term oncologic outcome were analyzed in relation to cN status in LARC patients treated by neoadjuvant CRT and prospectively followed up at a single institution.

For our patients treated with TME surgery after CRT, ypCR was achieved in 20.1 % of the cases, which is in line with the majority of studies previously reported in the literature.^5^ Our survival analysis supported the evidence of a favorable long-term oncologic outcome for patients displaying ypCR. In our series comparing ypCR patients with no-ypCR patients, the 5-year DFS rates were respectively 84.9 and 61.7 %. This is in line with the data reported by Maas et al.^9^ from a pooled analysis of 3105 LARC patients treated by preoperative CRT who showed a 5-year DFS of 83.3 % for ypCR patients compared with 65.6 % for no-ypCR patients. Similarly in a meta-analysis by Zorcolo et al.^41^ of 12 studies including 1913 LARC patients, the 5-year DFS was 86.9 % for ypCR patients compared with 63.9 % for no-ypCR patients. Recently Wasmooth et al.^42^ reported a 5-year DFS of 81 % for patients with ypCR and 50 % for patients without ypCR among 1384 patients enrolled in the national population-based colorectal cancer registry of Norway who had advanced T3 and T4 rectal cancer with N0-2,M0 managed by neoadjuvant long-course (chemo)radiation. Interestingly, ypCR was associated with a low risk of metastasizing.

In our subset of ypT0 patients treated by LE surgery, the local and distant recurrence rates were very low and similar to those for ypT0 patients treated by TME surgery. This finding is consistent with data reported by Borshitz et al.,^13^ who analyzed seven studies reporting oncologic outcome of LE after neoadjuvant CRT for cT2–3 tumors (*n* = 237). In their study, ypT0 was noted in 22 % of the cases, and the 5-year LRFS and DMFS were respectively 100 and 96 %. Similarly, Pucciarelli et al.^18^ reported that the 3-year LRFS was 96.9 % for 43 cT3 or low-lying cT2 rectal cancer patients treated with CRT followed by LE and observation for the ypT0-1 patients.

In our patients initially staged as cN+ and treated with CRT followed by TME surgery, metastatic lymph nodes at pathology were detected in 42.2 % of the cases. However, in the subgroup of patients with ypT0, metastatic lymph nodes were detected in 16 % of the surgical specimens. The rate of metastatic lymph nodes in LARC achieving ypT0 after CRT has been reported to vary between 2 and 17 %, which is in line with our findings of a 10 % rate (Table 2).^24^^–^ 33

**Table 2:** Metastatic lymph node rates in locally advanced rectal cancer with complete pathologic response in the primary tumor (ypT0) achieved after neoadjuvant chemoradiation

	No. of patients	Examined lymph nodes median (range)	YpT0		YpN+	
			*N*	%	*N*	%
Read et al.^24^	644	13 ± 8	42	6.52	1	2.38
Pucciarelli et al.^25^	235	9 (0–38)	56	23.83	1	1.79
Hughes et al.^26^	130	6 (0–21)	23	17.69	4	17.39
Guillem and Minsky^27^	188	9 (0–38)	37	19.68	1	2.70
Berho et al.^28^	86	13,1 (1–59)	18	20.93	2	11.11
Yeo et al.^29^	333 (all ypT0)	10 (0–78)	333	100	29	8.70
Jang et al.^30^	830	11	91	10.96	6	6.59
Tranchart et al.^31^	245	24 (3–60)	26	10.61	2	7.69
Park et al.^32^	725	11 (6–15)	143	19.72	13	9.09
Sprenger et al.^33^	398	28.0 ± 13.7	40	10.0	4	10.00
Current study	179	13 (2–37)	40	22.34	4	10.00

*ypT0* complete pathologic response in the primary tumor, *ypN* pathologic lymph node status

The assessment of response to treatment is becoming increasingly important in view of a personalized surgical approach. Among our patients, restaging accuracy using standard MRI and endorectal ultrasound was very low. This is in line with two recent meta-analysis leading to the conclusion that overall accuracy of restaging is not sufficiently consistent for clinical application.^34^,^35^ In addition, a nomogram using clinicopathologic parameters to predict ypN status after CRT developed in a training cohort of 891 LARC patients has been shown to achieve an accuracy of 0.77 in an external validation cohort of 258 patients.^36^

In view of the aforementioned limitations, even if surgical complications, including suture dehiscence and endoanal pain, are not uncommon among patients undergoing LE after CRT, as previously reported by us and others,^18^,^43^ this remains a procedure of investigational interest to confirm potential ypT0 status of patients with a major clinical response. On the other hand, a more conservative approach such as the “wait and see” option might be considered for patients with a complete clinical response.^44^ Hopefully, new techniques such as the fluorodeoxyglucose (FDG)-positron emission tomography (PET) scan and perfusion MRI might lead to a precise assessment of response.^45^–^48^

This study was limited by its single-center retrospective design and its small number of ypT0 patients displaying metastatic lymph nodes at pathology. In addition, the large time frame considered might have accounted for our relatively high local recurrence rate compared with the results of prospective clinical studies.

In conclusion, our findings indicate that treatment protocols aimed at organ preservation in rectal cancer achieving ypT0 after CRT can be offered also to patients with clinically positive mesorectal lymph nodes at their initial diagnosis. The favorable long-term outcome for ypT0 tumors and the risk of metastatic mesorectal lymph nodes should be discussed in patient–clinician communication.
